# Factors affecting quality of end-of-life hospital care - a qualitative analysis of free text comments from the i-CODE survey in Norway

**DOI:** 10.1186/s12904-020-00609-x

**Published:** 2020-07-07

**Authors:** Marit Irene Tuen Hansen, Dagny Faksvåg Haugen, Katrin Ruth Sigurdardottir, Anne Kvikstad, Catriona R. Mayland, Margrethe Aase Schaufel, on behalf of the ERANet-LAC CODE project group, Dagny Faksvåg Haugen, Katrin Ruth Sigurdardottir, Marit Irene Tuen Hansen, Karl Ove Hufthammer, Wojciech Leppert, Katarzyna Wolszczak, Eduardo Garcia Yanneo, Vilma Tripodoro, Gabriel Goldraij, Martin Weber, Christina Gerlach, Lair Zambon, Juliana Nalin Passarini, Ivete Bredda Saad, John Ellershaw, Grace Ting, Catriona Mayland, Anne Kvikstad, Eva Gravdahl, Julia Bratke, Janet Bakken, Kristin Vassbotn Guldhav

**Affiliations:** 1 Regional Centre of Excellence for Palliative Care, Western Norway, Haukeland University Hospital, Bergen, Norway; 2 Department of Clinical Medicine K1, University of Bergen, Bergen, Norway; 3 Department of Medicine, Haraldsplass Deaconess Hospital, Bergen, Norway; 4 Department of Cancer Research and Molecular Medicine, European Palliative Care Research Centre, Faculty of Medicine, Norwegian University of Science and Technology, Trondheim, Norway; 5 Palliative Medicine Unit, Cancer Clinic, St. Olavs Hospital, Trondheim University Hospital, Trondheim, Norway; 6 Department of Oncology and Metabolism, University of Sheffield, Sheffield, UK; 7 Palliative Care Institute, University of Liverpool, Liverpool, UK; 8 Department of Thoracic Medicine, Haukeland University Hospital, Bergen, Norway

**Keywords:** Palliative care, Cancer, Bereaved relatives, Death, Quality of healthcare, Survey, Free text comments, Qualitative research, Communication

## Abstract

**Background:**

The ERANet-LAC CODE (Care Of the Dying Evaluation) international survey assessed quality of care for dying cancer patients in seven countries, by use of the i-CODE questionnaire completed by bereaved relatives. The aim of this sub study was to explore which factors improve or reduce quality of end-of-life (EOL) care from Norwegian relatives’ point of view, as expressed in free text comments.

**Methods:**

194 relatives of cancer patients dying in seven Norwegian hospitals completed the i-CODE questionnaire 6–8 weeks after bereavement; recruitment period 14 months; response rate 58%. Responders were similar to non-responders in terms of demographic details.104 participants (58% spouse/partner) added free text comments, which were analyzed by systematic text condensation.

**Results:**

Of the 104 comments, 45% contained negative descriptions, 27% positive and 23% mixed. 78% described previous experiences, whereas 22% alluded to the last 2 days of life. 64% of the comments represented medical/surgical/oncological wards and 36% palliative care units. Four main categories were developed from the free text comments: 1) Participants described how attentive care towards the practical needs of patients and relatives promoted dignity at the end of life, which could easily be lost when this awareness was missing. 2) They experienced that lack of staff, care continuity, professional competence or healthcare service coordination caused uncertainty and poor symptom alleviation. 3) Inadequate information to patient and family members generated unpredictable and distressing final illness trajectories. 4) Availability and professional support from healthcare providers created safety and enhanced coping in a difficult situation.

**Conclusions:**

Our findings suggest that hospitals caring for cancer patients at the end of life and their relatives, should systematically identify and attend to practical needs, as well as address important organizational issues. Education of staff members ought to emphasize how professional conduct and communication fundamentally affect patient care and relatives’ coping.

## Background

Relatives who experience the death of a close family member possess unique perspectives on the patient’s suffering and needs [1]. These perspectives may provide access to prerequisites for tailored and effective medical treatment [2], and also challenge the views of healthcare professionals [3]. Thus, relatives play a central role both as caregivers and as participants in research illuminating how care for the dying person can be improved.

Ensuring high standards of care and support for patients dying from cancer and their relatives is of major global importance and relevance [4]. Good EOL care for cancer patients and their relatives entails a high degree of coordination and availability of healthcare services [5]. Earlier studies have also identified patients’ and families’ unmet needs regarding information and symptom relief [6], and how dignity at the end of life can be preserved by healthcare personnel addressing these aspects of care [7].

To improve the care, we need to be able to assess the current quality of care in a reliable manner. One internationally recognized method for evaluating care for dying patients is to ask bereaved relatives through post-bereavement surveys [8, 9]. ‘Care Of the Dying Evaluation’ (CODE™) is a recognised, validated post-bereavement questionnaire focused on both quality of patient care and support for the relatives in the patient’s last 2 days of life and the immediate post-bereavement period [8]. In addition to questions with pre-determined response categories asking about nursing and medical care, symptom relief, communication, emotional and spiritual support, and circumstances surrounding the death, responders may add free text comments. The free text comments are not limited to the last 2 days of life; on the contrary, responders are invited to comment on any aspect of care during the final illness trajectory.

The project ‘International Care Of the Dying Evaluation (CODE): quality of care for cancer patients as perceived by bereaved relatives’ (2017–2020) was funded by the Network of the European Union (EU) and the Community of Latin American States (CELAC) on Joint Innovation and Research Activities (ERANet-LAC) with the aim to advance the international evidence-base in care for the dying [10]. This involved undertaking an international survey of relatives to cancer patients dying in hospitals in seven countries across Europe and South America, by use of the international version of the CODE™ questionnaire [11]. In Norway, the call for improving end-of-life (EOL) care on all levels of the healthcare system has recently been outlined in a Norwegian Official Report (NOU) [12]. Research is warranted specifically addressing the user-perspective and how to optimise care during the palliative phase of incurable illness. The aim of the present study was to examine which factors improve or reduce quality of EOL care for cancer patients from Norwegian relatives’ point of view, by examining free text comments from the ERANet-LAC CODE international survey in Norway.

## Methods

### Design

A multicentre, post-bereavement observational study was conducted in bereaved family members of patients with cancer dying in the hospital setting, by use of the i-CODE questionnaire [11]. This qualitative sub study analyzed free text comments made by Norwegian participants, addressing aspects of care during the final illness trajectory.

### Setting and participants

Participants were next-of-kin to patients who had died on Medical, Surgical or Oncological wards or on Palliative Care units in seven hospitals in Norway. Inclusion and exclusion criteria are presented in Table 1, and apply both to the main study and the qualitative sub study. All free text comments were incorporated in the analysis.

**Table 1:** Inclusion and exclusion criteria of participants

**Inclusion criteria** - Relatives of patients with cancer dying an anticipated death in a hospital - 18 years or older - Being together with the patient in the hospital at least some of the patient’s last two days of life - Patient older than 18 years when he/she died - Patient being hospitalized for at least 3 calendar days - Capable of giving written informed consent, implicitly obtained by completing and submitting the questionnaire	
**Exclusion criteria** - Patient died suddenly and unexpectedly - Relatives were unable to complete questionnaire due to linguistic barriers or cognitive impairment - Staff assessed participation to be a huge burden for the relatives due to psychiatric illness or other severe condition	

The hospitals are representative of Norwegian hospitals in general, with respect to size, treatment levels and annual death rates. Demographic characteristics of patients and participants are presented in Table 2.

**Table 2:** Characteristics of deceased patients and bereaved relatives (*n* = 194)

	With free text comments from bereaved relatives (*n* = 104) n (%)^a^	Without free text comments from bereaved relatives (*n* = 90) n (%)^a^
**Patients**		
**Age** (years)		
18–29	1 (1)	0 (0)
30–39	1 (1)	3 (3)
40–49	7 (7)	5 (6)
50–59	13 (13)	13 (14)
60–69	30 (29)	25 (28)
70–79	34 (33)	31 (34)
≥ 80	17 (16)	11 (12)
NA	1 (1)	2 (2)
**Gender**		
Female	41 (39)	27 (30)
NA	1 (1)	2 (2)
**Cancer diagnosis**		
Breast	6 (6)	5 (6)
Gastrointestinal	37 (36)	29 (32)
Respiratory organs	27 (26)	19 (21)
Urological, incl. Prostate	10 (10)	16 (18)
Leukaemia and lymphoma	9 (9)	5 (6)
Other	17 (16)	21 (23)
**Died in a palliative care unit** **(PCU)**	37 (36)	41 (46)
**Specialist palliative care team involved (outside PCU)**	41 (39)	27 (30)
	mean (range) median	mean (range) median
**Length of last admission (days)**	12 (3–80) 9	11 (4–48) 9
	With free text comments (*n* = 104) n (%)^a^	Without free text comments (*n* = 90) n (%)^a^
**Bereaved relatives**		
**Age** (years)		
18–29	2 (2)	0 (0)
30–39	7 (7)	4 (4)
40–49	16 (15)	11 (12)
50–59	32 (31)	21 (23)
60–69	26 (25)	26 (29)
70–79	17 (16)	24 (27)
≥ 80	3 (3)	2 (2)
NA	1 (1)	2 (2)
**Gender**		
Female	70 (67)	66 (73)
NA	4 (4)	2 (2)
**Relationship to patient**		
Husband/Wife/Partner	60 (58)	59 (66)
Son/Daughter	35 (34)	19 (21)
Brother/Sister	4 (4)	5 (6)
Son−/Daughter-in-law	0 (0)	2 (2)
Parent	2 (2)	2 (2)
Friend	1 (1)	1 (1)
Other	1 (1)	1 (1)
NA	1 (1)	1 (1)
**Religious affiliation**		
Christian	70 (67)	76 (84)
Muslim	0 (0)	1 (1)
Hindu	1 (1)	0 (0)
Jewish	1 (1)	0 (0)
Any other religion	5 (5)	2 (2)
None	26 (25)	8 (9)
NA	1 (1)	3 (3)

^a^Due to rounding of decimals, all columns do not add up to exactly 100%. NA Not answered

### Data collection

The recruitment period lasted from August 15th 2017 to September 15th 2018 in five hospitals and from April to September 2018 in two hospitals. Eligible participants received written and oral information about the study from healthcare personnel before leaving the ward. Study information and the i-CODE questionnaire were sent by mail six to 8 weeks post bereavement to all eligible relatives who had not actively declined participation. One reminder was sent to non-responders. Relatives returned the questionnaire by mail in prepaid envelopes. Informed consent was implied when completing and submitting the questionnaire. The open responses were explicitly solicited and foreseen in the questionnaire.

### Analysis

Data analysis was performed by two of the authors (MITH and MAS) using systematic text condensation [13]. This analysis proceeds through the following steps: 1) Reading the material to obtain an overall impression, bracketing preconceptions, 2) identifying units of meaning, representing different aspects of the participants’ EOL care experiences and coding for these, 3) condensing and abstracting the meaning within each of the coded groups, and 4) summarizing the contents of each coded group to generalized descriptions and concepts reflecting the most important factors influencing EOL care, as perceived by the participants. Categories and findings were developed from the empirical data using an editing analysis style as described by Miller and Crabtree [14]. All comments were also sorted as positive, negative or mixed descriptions.

## Results

### Participants

One hundred and ninety-four bereaved relatives completed and returned the survey, (194/334, response rate 58%). One hundred and four of these (58% spouse/partner) added free text comments. Most of the 104 participants belonged to the age group 50 to 69 years, 67% reported a Christian religious affiliation, and 39% were women.

### Free text comments

Of the 104 comments, 45% contained negative descriptions, 27% positive and 23% mixed. 78% described previous experiences, whereas 22% alluded to the last 2 days of life (Table 3).

**Table 3:** Characteristics of free text comments (*n* = 104)

	n (%)
Negative descriptions	47 (45)
Positive descriptions	28 (27)
Mixed descriptions (both positive and negative)	24 (23)
Other comments (related to the questionnaire itself)	5 (5)
Describing only the last two days	23 (22)
Describing experiences before the last two days	81 (78)
From palliative care units	37 (36)
From medical/surgical wards	46 (44)
From oncology wards	21 (20)

Four main categories were developed from the free text comments: 1) Participants described how attentive care towards the practical needs of patients and relatives promoted dignity at the end-of-life, which could easily be lost when this awareness was missing. 2) They experienced that lack of staff, care continuity, professional competence or healthcare service coordination caused uncertainty and poor symptom alleviation. 3) Inadequate information to patient and family members generated unpredictable and distressing final illness trajectories. 4) Availability and professional support from healthcare providers created safety and enhanced coping in a difficult situation. Below, we elaborate on these findings.

### Attentive care towards practical needs promoted dignity at the end of life

Several participants described the importance of practical needs being met by healthcare personnel, e.g. allowing them to sleep in the patient’s own room, or facilitating meaningful activities such as playing music, lighting candles and being together day and night. They valued the opportunity to be present and close to their family member as much as possible. Feeling welcome and allowing unlimited access made it easier for them to care and support the patient. This was also deeply appreciated at earlier stages of the illness trajectory when the patient’s condition worsened, not only during the terminal phase.

**“*Fortunately, almost everyone was very positive, caring, and brought newspapers, magazines, coffee, and biscuits. A pat on the back, encouraging words, the little extra things that made it easier for us to be relatives. We were allowed to stay there all the time, night and day, which lightened the situation both for us and for her.” (Informant 37).***

Other participants outlined how they missed practical help supplying food and drink during hospital stays with their relatives in the last phase of life. They described how they either had to leave the patient to obtain a meal, or ask friends to come by with something to eat. Facilities such as free parking were also needed. Participants emphasised how they did not want to go away from their family member and that practical issues then became a burden. Being terminally ill in a room shared with other patients deprived dignity from their next of kin. In the terminal phase of the patients’ illness, discussions about potential transfers to other units in the hospital or to the community setting, were experienced as distressing. It troubled them and gave the impression that they themselves, as well as the patient, were an inconvenience to the staff and a perceived burden to the hospital.

***“As a relative, it is both an unpleasant and very sad experience to feel that your closest family member is a nuisance to the hospital. Choices made by the hospital may convey respect and dignity – or the opposite.” (Informant 82).***

Some participants also described how essential a calm and peaceful environment was to their overall experience of the last phase of their family member’s life. One of them elaborated on how noise from another family’s expressive grieving following another patient’s death in the room next door installed fear and sadness to their own dying relative and themselves. No explanation or support was given by the staff, and this experience haunted them for quite a while, reducing dignity and comfort at the end of life.

### Insufficient healthcare services caused insecurity and poor symptom alleviation

The consequences of staff shortage were described as compromising safety and treatment. Participants explained how they were fearful of leaving their family members, if they were weak or had difficulty standing up, mindful of the risk of falls on the ward. They also tried to be present during meals in order to assist family members who were having difficulty eating patients. The regular practice of reduced staff numbers during weekends was experienced as hazardous. Participants felt responsible for observing their family member patient and reporting to the staff if they perceived that medication was needed to help with pain control or restlessness. They did not feel comfortable in this role, and suspected that their uncertainty might have delayed pain relief.

***“There were way too few people at work. As relatives, we didn’t dare to leave him. We had to look after him ourselves all the time and tell which medication he needed.” (Informant 103).***

Lack of continuity among doctors was another important aspect affecting their overall experience and treatment. There were many different doctors involved in the provision of care both in the outpatient clinic and during hospital admissions, meaning frequent re-telling of the history. It was regarded as very disturbing when a doctor, whom patients and families had not previously met, imparted new and crucial information. Continuity was asked for both during initial investigations and the following treatment phase, as well as coordination of care instead of fragmentation. The latter could create a feeling of chaos where no one actually had overall control, expressed in strong wordings such as “15 months of mess” and “too many cooks spoil the broth”. Uncertainty remained about how much cooperation occurred between different medical specialists and their family doctor.

***“The illness history had to be told over again at every visit, and there was no coordination of care between the general practitioner, the doctor at the local hospital and the university clinic.” (Informant 88).***

Lack of competence could also deeply affect participants’ experience of the quality of care at the end of life. Some described how inexperienced nurses administered pain medication but left the room in a hurry without addressing other needs, e.g. having a conversation about the challenging situation. Another participant outlined how their family member being confused and helpless due to the underlying disease did not receive respectful and adequate care, interpreted by the relatives as relating to lack of knowledge among staff. These family members missed being on a palliative care unit and desired an increased competence of staff who care for dying patients. The organization of care through the final illness for cancer patients was also questioned.

***“My husband was admitted for pain relief. He was in a lot of pain, and the staff didn’t manage to alleviate his suffering. He should have been admitted to a palliative care unit. It’s impossible to understand why a cancer patient at the end of life must be admitted to the department responsible for the initial tumour treatment.” (Informant 63).***

### Inadequate information generated unpredictable and distressing illness trajectories

Participants wanted to know more about what the last days of life would be like, and felt they themselves often had to request this information. They missed critical information during initial discussions, or needed to have this repeated. Some thought doctors avoided giving honest information, and although they could appreciate the challenges when delivering bad news, most preferred to have clear-cut information. It was difficult for them to understand what was going on with their family member, for instance, when intravenous fluids had been discontinued. The lack of explanations caused additional distress. They also underlined the importance of an update on the latest development in between visits. Even when the patient’s condition worsened and imminent death was suspected, information could still be delayed and unclear, expressed as “beating about the bush”. Not knowing that the patient was approaching a terminal phase could deprive them from precious time together.

***“I was not adequately informed that the end was near during his last days of life. Had I known, I could have been present the last hours he had left to live, but unfortunately, that was not the case. I am being haunted daily by the fact that I was not there when he died.” (Informant 26).***

Participants also described receiving too little information upon discharge. Some explained how they were told their next of kin would die, but missed emotional support. Written information could be of help, but could not replace supportive conversations for building trust and gaining clarifications. Participants stated they had important knowledge about the patient that was not asked for, and wished they could have been more involved. It was perceived as a huge burden when they met healthcare professionals who did not understand their wishes and needs e.g. place of care, level of treatment. Several viewed the time from the last cancer treatment until the terminal phase to be the most difficult period, in which they felt abandoned.

***“We were left all by ourselves after the last chemotherapy, and wished we had further follow-up. We felt all alone and had to contact the hospital ourselves upon demand. The time from the last treatment until the last two weeks of life was the hardest period for us.” (Informant 99).***

### Professional support from healthcare providers created safety and enhanced coping

Other participants expressed deep gratitude and solely positive feelings in their feedback to the hospital staff. It was clear that easy access to advice from healthcare professionals whom they knew, provided great comfort and support. Availability and time to explain all aspects of a matter conveyed empathy and security that enabled relatives to cope with the situation. Being treated in a respectful, professional manner created a trusting and confident relationship with the doctor and nurses in charge. Professionalism encompassed willingness to elaborate information to the extent that every family member involved understood what was at stake. This included giving priority to and space for important conversations with children and grandchildren. Participants noticed how staff managed to remain professional and compassionate despite limited resources, and went above and beyond expectations to meet the needs of patients and their families.

***“I think nurses deserve all the respect they can get. They perform their duties in a professional manner and at the same time act incredibly humane. To me as a son I felt that the nurses conveyed real tenderness and understanding when helping the patient.” (Informant 65).***

The opposite experience was described amongst those who faced episodes of professional misconduct in terms of insensitive communication, unsympathetic gestures or broken promises. They pointed at incidents that may have been details or involuntary violations for staff but ended up adding a further burden to their strain. Some had been told they would receive a phone call from the hospital department after the death, but never received it. Others sensed a lack of empathy and support when being informed that there was nothing more that could be done for their family member. Relatives and patients felt insecure when the doctor was not properly prepared for a consultation or forgot appointments. The impact of wording and mode of expression was emphasised:

***“Nurses need to be more aware of how they express themselves regarding family members’ choices. I was extremely tired the last week before my sister’s death and needed to go home and rest. For about three weeks, hospital staff who assessed she was dying, regularly called me, and when I needed a break, the attending nurse said I ought to stay. Yet she didn’t die. Relatives need acceptance when sleep is required!” (Informant 89).***

## Discussion

Our findings add new knowledge describing how lack of information may lead to more distressing and unpredictable illness trajectories both for patients and their relatives. The results illuminate how practical needs can generate dilemmas and strain when family members themselves have to leave the patient for food or rest, or experience undignified settings. This underlines the importance of paying attention to the “small things” that matter most [15]. The detailed descriptions of how organizational aspects of EOL care influenced symptom relief and relatives’ support expand our understanding of how crucial these conditions are for adequate treatment and care. We believe our results can be used by healthcare managers when improving services in their institutions.

Participants wished healthcare personnel to be both professional and compassionate. Relatives associated professionalism with good skills both in terms of delivering high standard care as well as adequate information. Compassion is linked to empathy and ranked by patients and family members to be among their most important healthcare needs, yet difficult to define [16]. This understanding of professionalism presupposes a distinction between these two attributes, which is challenged by Nortvedt and Nortvedt [17]. They argue that professionalism in healthcare does not necessarily need to be associated with an objective distance, which seems incompatible with care and proximity. On the contrary, clinical professionalism requires sensitivity and thoughtfulness as key elements of the healthcare personnel-patient-relationship. Factors that may foster or inhibit an appropriate understanding and communication of the patient’s experiences are important to acknowledge during healthcare professional education [18]. A biomedical approach for instance to the patient’s pain, is also needed in order to provide adequate symptom relief while maintaining the necessary professional distance. Yet this does not rule out the will to get involved in patients’ personal setting, which is so deeply appreciated by our participants. Getting to know the staff and building trust in a vulnerable situation were crucial for their assessment of quality care. Being aware of the relational reality of care may challenge the traditional ideal of detachment in the medical encounter, avoiding situations in which patients may be “harmed” in the absence of care [19].

Learning professionalism is closely linked to developing an identity as healthcare personnel. Professional identity may be described as a representation of self, achieved in stages over time during which the characteristics, values, and norms of the individual thinking, acting and feeling like a physician or nurse are formed [20]. This process is increasingly addressed and debated in medical education and training, including Norway [21]. Nurses have traditionally had a greater theoretical understanding and increased focus on how their professional conduct and identity are linked to the quality of care that is delivered [22]. Our findings suggest that institutions caring for patients and their relatives at the EOL should have an increased attention towards and measures for adequate training of their staff in these matters. A special focus is needed on preserving dignity at the end of life, as described by Chochinov [23]. His model incorporates attitude, behavior, compassion and dialogue as the main areas in which healthcare professionals can ensure patients’ dignity, and describes in detail how to achieve the required skills [24]. Another perspective is added by Allmark, who draws a distinction between “death with dignity” and “death without indignity” [25]. Using this distinction, the responsibility of healthcare professionals is to ensure indignities are minimized, e.g. controlling pain and respecting patients’ decisions and wishes.

Palliative care encompasses family members in a particular way that increases quality of care [26]. Acknowledgement of the importance of the healthcare professional-family carer relationship is pertinent, due to the potential impact on both perceptions of patient care and the subsequent grieving process [27, 28]. Our participants imparted how they had vital and relevant knowledge about the patient’s needs, knowledge which was not asked for or taken into account, as also reported in other studies [29, 30]. This omission diminished the quality of care of the terminal illness trajectory in terms of inadequate symptom relief as well as unmet wishes about place of care. Relatives also experienced this lack of interest as hurtful and insensitive. Comprehensive communication skills should be strived for in all settings treating patients in need of palliative care [31, 32]. As modern hospital strategies emphasize user perspectives to be incorporated into care, there is a need to further formalize and ensure this development [33].

## Strengths and limitations

Free text comments are used to supplement standardized questionnaires with additional perspectives participants might have on the topic of investigation, including in the palliative care setting [28]. It is recognized that informants having very good or very poor experiences are more likely to provide qualitative free-text responses [34]. Even so, valuable nuances can be distinguished which otherwise would have been missed in solely quantitative data collection [32, 35]. Our study comprised a representative sample of both participants and hospitals in Norway, adding transferability to our findings. Few informants reported another religious affiliation than Christianity, making it less likely that the results will be transferable to a different cultural and religious setting. We obtained a representative sample of patients and relatives in palliative cancer care, as gastrointestinal and lung cancer accounted for the majority of deaths, and most patients were older than 60 years. There were no major differences between the group adding free text comments and the group with no comments. Only 22% of the comments described aspects of care during the last 2 days of life, which was the main focus of the i-CODE study. Thus, our findings describe factors of EOL care that participants regarded as important over a longer time span.

The majority of comments belonged to relatives of patients dying outside palliative care units. We have not performed separate qualitative analysis in the different subgroups and are unable to compare the results across units. However, differences between subgroups will be analyzed in the main quantitative study. We do not know for sure if the reference relative in the hospital was the same as before admission, but the questionnaire was sent to the person designated as “main relative” in the hospital records. Data regarding length of illness and degree of information received by the patient and relatives about the illness were not collected, except from knowing it was cancer with short life-expectancy.

Analysis was conducted in collaboration between two of the authors, a nurse (MITH) and a physician (MAS). Our clinical background from palliative care in the nursing home setting (MITH) as well as thoracic medicine and cardiology (MAS) provided a broad framework for discussions on analytical choices but also influenced which findings to be emphasized. The other authors, being experienced oncologists and palliative care specialists, critically challenged and revised the categories to clarify perspectives and presuppositions.

## Conclusions

Our findings suggest that hospitals caring for cancer patients at the end of life and their relatives, should systematically identify and attend to practical needs, as well as address important organizational issues. Education and training of staff members ought to emphasize how professional conduct and communication fundamentally affect patient care and relatives’ coping.

## Data Availability

Access to the dataset is restricted, but may be considered upon reasonable request to the corresponding author.

## Abbreviations

EOL: End-of-life
CODE: Care Of the Dying Evaluation
